# Isolation and identification of membrane vesicle-associated proteins in Gram-positive bacteria and mycobacteria

**DOI:** 10.1016/j.mex.2014.08.001

**Published:** 2014-08-19

**Authors:** Rafael Prados-Rosales, Lisa Brown, Arturo Casadevall, Sandra Montalvo-Quirós, Jose L. Luque-Garcia

**Affiliations:** a Department of Microbiology and Immunology, Albert Einstein College of Medicine, Bronx, NY 10461, USA; b Department of Analytical Chemistry, Complutense University of Madrid, Madrid 28040, Spain

**Keywords:** Gram positive bacteria, Membrane vesicles, Protein content, Vesicles isolation

## Abstract

Many intracellular bacterial pathogens naturally release membrane vesicles (MVs) under a variety of growth environments. For pathogenic bacteria there are strong evidences that released MVs are a delivery mechanism for the release of immunologically active molecules that contribute to virulence. Identification of membrane vesicle-associated proteins that can act as immunological modulators is crucial for opening up new horizons for understanding the pathogenesis of certain bacteria and for developing novel vaccines. In this protocol, we provide all the details for isolating MVs secreted by either mycobacteria or Gram-positive bacteria and for the subsequent identification of the protein content of the MVs by mass spectrometry. The protocol is adapted from Gram-negative bacteria and involves four main steps: (1) isolation of MVs from the culture media; (2) purification of MVs by density gradient ultrucentrifugation; (3) acetone precipitation of the MVs protein content and in-solution trypsin digestion and (4) mass spectrometry analysis of the generated peptides and protein identification. Our modifications are:

• Growing Mycobacteria in a chemically defined media to reduce the number of unrelated bacterial components in the supernatant.
• The use of an ultrafiltration system, which allows concentrating larger volumes.
• In solution digestion of proteins followed by peptides purification by ziptip.

## Graphical abstract

**Figure:**
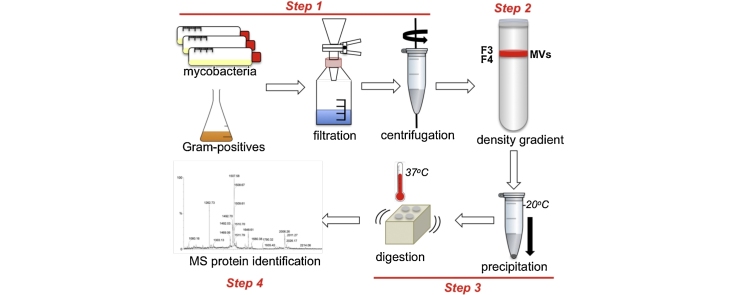

## Method details

Isolation of pure mycobacterial and Gram-positive bacteria (*Bacillus subtillis*) membrane vesicles involves growing the bacteria in liquid media to mid-logarithmic phase, followed by clarification of the cell supernatant by sequential filtration and concentration. The concentrate is then ultracentrifuged and the pellet is submitted to density gradient ultracentrifugation to obtain the pure membrane vesicle fractions [1–3]. Proteins in the purified fractions are then acetone precipitated and in-solution digested with trypsin. Generated peptides are separated by nano-LC and analyzed by mass spectrometry for protein identification [4,5].

### Step 1: isolation of membrane vesicles

#### Materials

• Minimal media (MM): Minimal media or chemically defined media is used to reduce the number of components in the culture supernatant. To approximately 3.9 L of double distilled water in a 4 L beaker, add 4 g KH_2_PO_4_, 10 g Na_2_HPO_4_ and 2 g asparagine. Adjust the pH to 7.0 using HCl. Filter-sterilize (0.22 mm) the solution. Add 56 ml of filter-sterilized 50% (v/v) glycerol, and 0.4 ml of filter-sterilized 500 mg/l ferric ammonium citrate, and 0.4 ml of 5 mg/l CaCl_2_, and 0.4 ml of filter-sterilized 1 mg/l ZnSO_4_.
• 7H9 media: To 450 ml of double distilled water in a 500 ml beaker, add 2.35 g of 7H9 Middlebrook broth. When dissolved, filter-sterilize (0.22 μm) the solution and add 50 ml of the sterile supplement OADC.
• Brain Heart Infusion (BHI) broth and agar (Difco).
• Luria Bertani (LB).
• Plastic squared inkbottles (30 ml).
• Orbital shaker 90 rpm at 37 °C.
• Roller bottles 850 cm^2^ (Corning) equipped with a membrane on the cap.
• Rack with a horizontal rolling motion (30 rpm) at 37 °C.
• Vacuum filtration units (1000 ml, low protein binding) equipped with 0.45 μm and 0.22 μm PVDF membrane.
• Table top ultracentrifuge, rotor and 3.3 ml tubes with capacity to spin at 100,000 × *g*.
• Amicon ultrafiltration system with a 100-kDa-exclusion filter.

*Note*: This list includes only non-standard items. Generic components are assumed to be available.

#### Procedure

1. For mycobacteria, start a culture in 10 ml of 7H9 media. Carefully thaw the mycobacterial glycerol stock and add it to the 10 ml of 7H9 media in a 30 ml square inkbottle. Incubate the culture at 37 °C in an orbital shaker at 90 rpm for 7 days. For fast-growing mycobacterial strains (*Mycobacterium smegmatis*), incubate the culture at 37 °C in an orbital shaker at 90 rpm for 2 days. For Gram-positive bacteria, inoculate the glycerol seed in 10 ml of BHI or LB and incubate at 37 °C in an orbital shaker at 200 rpm for 15–18 h.
2. For mycobacteria, prepare 4 L of MM and distribute the media into 8 roller bottles (500 ml each). For Gram-positive bacteria prepare 200 ml of BHI or LB and transfer to a 1 L flask.
3. Inoculate 1.25 ml of 7H9 mycobacterial culture into roller bottles containing 500 ml of MM. For Gram-positives inoculate 1 ml of the BHI or LB preculture into the 200 ml of fresh BHI or LB.
4. For mycobacteria, incubate the bottles at 37 °C for 14 days with a rolling motion (30 rpm). For Gram-positive bacteria incubate the flask at 37 °C overnight (∼18 h) in an orbital shaker at 200 rpm.
5. Filter the cultures through a 0.45-μm-pore size filter.
6. Filter the clarified supernatants through a 0.22-μm-pore size filter.
7. Concentrate the supernatant using an Amicon Ultrafiltration system with a 100-kDa-exclusion filter. Leave 10–15 ml of liquid.
8. Rinse the filter with the remaining supernatant and recover the concentrate.
9. Sequentially centrifuge the concentrate at 4000 and 15,000 × *g* (15 min, 4 °C) to remove aggregates.
10. Ultracentrifuge the remaining supernatant at 100,000 × *g* for 1 h at 4 °C to obtain the membrane vesicle pellet.

### Step 2: purification of membrane vesicles by density gradient ultracentrifugation

#### Materials

• OptiPrep^®^ solutions: Prepare solutions of 30, 25, 20, 15 and 10% (v/v) diluting a 60% OptiPrep^®^ stock solution in DPBS.
• DPBS (Dulbecco's phosphate buffered saline).
• Ultracentrifuge, swinging rotor and 12.5 ml clear tubes, with capacity to spin at 140,000 × *g*.

#### Procedure

1. Discard the supernatant and suspend the pellet in 1 ml of DPBS, and mixed with OptiPrep^®^ solution (Sigma–Aldrich) to obtain a 35% [v/v] OptiPrep^®^ solution (final concentration).
2. Transfer to a 12.5 ml clear ultracentrifuge tube.
3. Overlay the vesicle sample with a series of OptiPrep^®^ gradient layers with Optiprep concentrations ranging from 10 to 35% (w/v).
4. Ultracentrifuge the gradient at 140,000 × *g* for 16 h at 4 °C using a swinging rotor.
5. Take 1 ml fractions from the top of the gradient to the bottom and keep the 3 and 4 fractions, which contain the pure membrane vesicles. If it is the first time isolating membrane vesicle from a bacteria it is recommended to analyze each fraction for the presence of membrane vesicles. We recommend submitting samples to electron microcopy analysis, performing an analysis by immunoblot with specific antibodies against vesicle-associated proteins, or metabolic labeling of vesicles to identify the radio-labeled vesicle fractions.
6. Pool the selected vesicle-containing fractions.
7. Dilute the pooled fractions in DPBS and centrifuge at 38,400 × *g* for 2 h to remove the Optiprep.
8. Suspend the vesicle pellet in 0.5 ml of DPBS.

### Step 3: Protein digestion

As with any sample preparation method for MS analysis, special attention must be paid to avoid loss or contamination of the samples during processing. Please be very careful not to use bare hands, loose hair, dirty glass- and plastic-ware, and always wear gloves (powder free and rinsed with water and ethanol before use) to minimize contamination by keratins, or these proteins will overwhelm low level protein samples and preclude successful analysis of the proteins of interest.

#### Materials

• Mass Spec-grade acetone at −20 °C.
• 25 mM ammonium bicarbonate: dissolve 0.2 g of ammonium bicarbonate in 100 ml of Mass Spec-grade water.
• 10 mM DTT: dissolve 15 mg of dithiothreitol (DTT) in 10 ml of 25 mM ammonium bicarbonate.
• 10 mM iodoacetamide: dissolve 18 mg of DTT in 10 ml of 25 mM ammonium bicarbonate.
• 50 ng/μl trypsin solution: dissolve 100 μg of trypsin gold, mass spectrometry grade (Promega), in 2 ml of 25 mM ammonium bicarbonate.
• Microcentrifuge with speed up to 13,000 rpm.
• Thermomixer with 1.5 ml tubes block (Eppendorf).
• Centrifugal vacuum concentrator with a rotor to accommodate 1.5 ml tubes.

*Note*: All solutions and buffers should be prepared in Mass Spec-grade water. Reconstituted trypsin can be stored at −20 °C for up to 1 month. For long-term storage, freeze reconstituted trypsin at −70 °C. Before use, thaw the reconstituted trypsin at room temperature, placing on ice immediately after thawing. To maintain sufficient enzymatic activity, limit the number of freeze-thaw cycles to 5.

#### Procedure

1. Add 6 volumes of ice-cold acetone to the vesicle pellet suspended in DPBS and incubate overnight at −20 °C.
2. Centrifuge at 4 °C during 10 min at 13,000 rpm. Remove supernatant very carefully and discard.
3. Wash the precipitate with 100 μl of ice-cold acetone. Repeat the centrifugation at 4 °C for 10 min at 13,000 rpm and discard supernatant.
4. Resuspend the pellet in 100 μl of 25 mM ammonium bicarbonate and transfer to a 200 μl tube (PCR style).
5. Add 10 μl of DTT solution. Incubate 1 h at room temperature for reducing the disulfide bonds of proteins.
6. Add 10 μl of idoacetamide solution and incubate 1 h at room temperature in the dark. Iodoacetamide binds covalently with the thiol group of cysteines, thus avoiding formation of disulfide bonds.
7. Add 40 μl of trypsin solution and incubate at 37 °C overnight (∼16 h) with gentle agitation using a thermomixer at 300 rpm. Cap the tubes tightly and cover with parafilm to avoid evaporation.
8. After completing the digestion, dry the samples using a centrifugal vacuum concentrator.

### Step 4: peptide analysis and protein identification

The following instructions assume the use of a NanoLC Plus (Eksigent) HPLC system coupled directly to an LTQ XL linear ion trap mass spectrometer (ThermoFinnigan). Similar instruments can also be used.

#### Materials

• C_18_ ZipTip^®^ 10 μl pipette tips (Millipore).
• Buffer A: 2% Mass Spec-grade acetonitrile, 0.1% formic acid in Mass Spec-grade water.
• Buffer B: 0.1% formic acid in Mass Spec-grade acetonitrile.
• NanoLC system coupled to a biological mass spectrometer able to perform MS/MS analysis.
• Database engine such as Mascot (Matrix Science) for protein identification.

#### Procedure

**Fig. 1:**
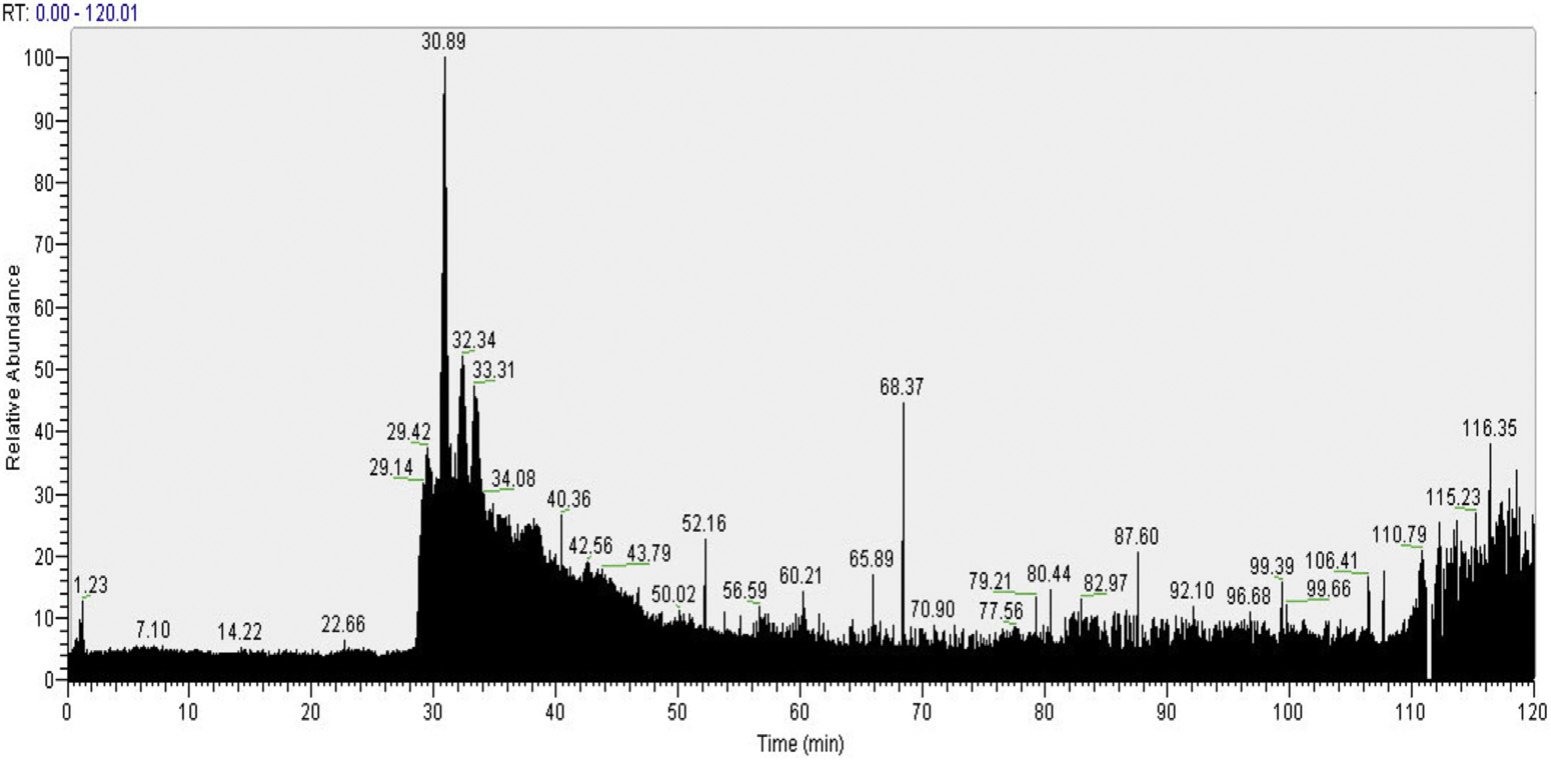
Typical chromatogram obtained with the described system. Peptides are eluted with a gradient starting at 95% buffer A and ending at 90% buffer B. The gradient is delivered over 120 min at a flow-rate of 200 nl/min.

1. Resuspend the dried peptides in 10 μl of buffer A and sonicate 10 min in an ultrasonic bath.
2. Use ZipTip^®^ for desalting the solution according to the manufacturer's instructions.
3. Elute peptides with 5 μl of buffer A and transfer the solution to a sample vial compatible with the autosampler of the liquid chromatograph.
4. Load the peptides onto a 0.3 mm × 5 mm C18 precolumn (New Objective) at a flow-rate of 3 μl/min.
5. Reverse the flow through the precolumn and elute the peptides with a gradient starting at 95% buffer A and ending at 90% buffer B. The gradient is delivered over 120 min at a flow-rate of 200 nl/min through a 75 mm × 15 cm fused silica capillary C18 HPLC column (LC Packings) to a stainless steel Nanobore emitter (Proxeon). The electrospray needle can vary depending on the ion source of the mass spectrometer. Fig. 1 shows a typical chromatogram obtained with the described system.
6. Use a database search engine such as Mascot (Matrix Science) for database searching and protein identification.
